# Liquid–Liquid Flows with Non-Newtonian Dispersed Phase in a T-Junction Microchannel

**DOI:** 10.3390/mi12030335

**Published:** 2021-03-22

**Authors:** Anna Yagodnitsyna, Alexander Kovalev, Artur Bilsky

**Affiliations:** 1 Kutateladze Insitute of Thermophysics SB RAS, 630090 Novosibirsk, Russia; therfmig@gmail.com (A.K.); bilsky@itp.nsc.ru (A.B.); 2 Physics Department, Novosibirsk State University, 630090 Novosibirsk, Russia

**Keywords:** microfluidics, liquid–liquid flow, shear-thinning fluid, flow pattern, slug flow

## Abstract

Immiscible liquid–liquid flows in microchannels are used extensively in various chemical and biological lab-on-a-chip systems when it is very important to predict the expected flow pattern for a variety of fluids and channel geometries. Commonly, biological and other complex liquids express non-Newtonian properties in a dispersed phase. Features and behavior of such systems are not clear to date. In this paper, immiscible liquid–liquid flow in a T-shaped microchannel was studied by means of high-speed visualization, with an aim to reveal the shear-thinning effect on the flow patterns and slug-flow features. Three shear-thinning and three Newtonian fluids were used as dispersed phases, while Newtonian castor oil was a continuous phase. For the first time, the influence of the non-Newtonian dispersed phase on the transition from segmented to continuous flow is shown and quantitatively described. Flow-pattern maps were constructed using nondimensional complex We^0.4^·Oh^0.6^ depicting similarity in the continuous-to-segmented flow transition line. Using available experimental data, the proposed nondimensional complex is shown to be effectively applied for flow-pattern map construction when the continuous phase exhibits non-Newtonian properties as well. The models to evaluate an effective dynamic viscosity of a shear-thinning fluid are discussed. The most appropriate model of average-shear-rate estimation based on bulk velocity was chosen and applied to evaluate an effective dynamic viscosity of a shear-thinning fluid. For a slug flow, it was found that in the case of shear-thinning dispersed phase at low flow rates of both phases, a jetting regime of slug formation was established, leading to a dramatic increase in slug length.

## 1. Introduction

Microfluidic technology in conjunction with gas–liquid and liquid–liquid flows have shown significant advances in chemical-reaction engineering and other applications at the microscale. Microfluidic extractors [1], mixers [2], and heat exchangers [3] were proved to intensify heat and mass transfer rates compared to conventional large-scale devices. Generation of microdroplets using microfluidic T-junctions or flow-focusing inlets allows quick production of uniform emulsions [4]. At the same time, small-scale flows in microchannels are convenient for precise control over biological objects when sorting and handling them [5].

The key feature of microfluidic devices is an extremely high surface-to-volume ratio, which provides most of the listed applications. However, since gravity and inertia become negligible with a decrease in size, new issues are introduced in the fluid flow. Capillarity and adhesion dominate the two-phase microfluidic systems and add complexity due to contact lines and interfaces. Further sophistication is superimposed if one of the phases possesses non-Newtonian properties. Many fluids used in technological processes, such as biological fluids in organ-on-a-chip systems [6]; bio-microelectromechanical systems (bio-MEMS), including plasmonic and electrochemical biosensors [7]; and polymer solutions in a number of chemical technologies [8] exhibit non-Newtonian properties. The design and optimization of such microchannel devices require fundamental knowledge about the flow regimes implemented in them and the physics of the processes occurring in this case. Despite non-Newtonian fluids being ubiquitous in nature and man-made devices [9], their flows in microgeometries remain poorly understood. The questions regarding flow-pattern-map unification, optimal droplet-generation regimes, and calculation of effective shear rates in two-phase flows at microscale are still open.

Microfluidics was intensively studied during the last two decades in order to understand underlying phenomena in liquid–liquid and gas–liquid flows. Different flow patterns are formed depending on flow velocities and properties of materials. Parallel or annular, plug (also known as a slug or Taylor flow), dispersed (droplet), and some accessory types of flow patterns are usually observed by researchers in microfluidic two-phase flows [10,11]. The onset of a particular flow pattern can be explained by the forces prevailing in the system for given values of problem variables. Dimensional analysis performed in a series of previous works revealed that the force balance for considered systems can be expressed in terms of several dimensionless groups, namely Reynolds number (Re), Weber number (We), capillary number (Ca), and Ohnesorge (Oh) or Laplace number (La) for each phase. Some authors also consider dimensionless ratios such as viscosity ratio λ = μ_d_/µ_c_, rectangular channel aspect ratio β = h/w, etc. These numbers and their combination in certain powers were used for flow-pattern-map construction and unification. Waelchli et al. [12] proposed the following composite numbers for flow-pattern maps of gas–liquid flows in rectangular microchannels: A·Re^α^We^β^(k_s_/D_h_)^γ^ for continuous phase and Re^α^We^β^ for dispersed phase, where k_s_/D_h_ is a ratio of channel roughness to the hydraulic diameter; A is a constant; and α, β, and γ are fitting parameters [12]. They obtained the best data collapse for values of α = 0.2, β = 0.4, γ = 5, and A = 10^7^. Similar dimensionless groups based on Re and We numbers, but without consideration of channel roughness, were obtained and tested against different flow conditions in [13]. For liquid–liquid flows, the authors obtained negative We number powers. Other variations of the composite dimensionless group were proposed in [14,15]. Yagodnitsyna et al. obtained a dimensionless group that consisted of We and Oh numbers [16]. This group yields a good unification for different liquid–liquid sets in a specified geometry, and modifications of powers to We^0.4^Oh^0.6^ provide an almost perfect prediction for a transition between parallel and segmented flow patterns in the case of different viscosity ratios [17].

Though the use of non-Newtonian fluids is expected to impact flow-pattern transitions, only a few investigations have been performed considering the influence of non-Newtonian properties on flow-pattern maps. Yang and colleagues studied flow patterns of several gas–liquid sets with a non-Newtonian continuous phase in microchannels with different geometry [18]. A new flow pattern called chained bubble slug was observed for a polyacrylamide (PAM) aqueous solution due to its high elasticity. The flow-pattern maps constructed in terms of phase superficial velocities were strongly influenced by fluid rheology. Transition boundaries were offset for different non-Newtonian solutions, and a large deviation from existing models was reported. The related work on gas–liquid flows was performed by Zhang et al. [19]. They used carboxymethyl cellulose (CMC) and sodium dodecyl sulfate (SDS) solutions as a continuous phase and nitrogen as a dispersed one. They found poor agreement when testing experimental data against existing models. A model was proposed based on Re, Ca, viscosity ratio, and surface roughness. The model gave better data collapse, although the usage of surface roughness as a parameter is a questionable approach in the case of the channels made of the same material. Fu et al. [20] conducted experimental research on liquid–liquid flow patterns in CMC aqueous solutions and Newtonian cyclohexane. They found droplet flow to occur at a lower superficial velocity of the continuous phase and parallel flow at a lower superficial velocity of the dispersed phase for higher concentrated CMC aqueous solutions. Flow-pattern maps were compared against existing dimensionless groups, including those by Waelchli et al. and Zhang et al.; however, map unification appeared to be insufficient. The following dimensionless combination was proposed: A·We^α^(H/W)^β^(k_s_/D_h_)^γ^(µ_c_/μ_d_)^δ^, which takes into account the rectangular channel aspect ratio H/W. Interestingly, this model seems to be strongly dependent on the viscosity ratio of phases, the aspect ratio, and the dimensionless roughness of the channel, rather than the Weber number effects.

Several research groups investigated the formation of plugs and droplets with either dispersed or continuous phases that were non-Newtonian. Comprehensive research on the formation of non-Newtonian droplets in a T-junction was presented by Husny et al. [21]. They proposed a model for droplet-diameter prediction, taking into account the viscosity ratio of liquid using a modified Ca number. Filaments turning into the secondary droplet were observed for droplets of non-Newtonian fluids at the formation stage. However, the presence of elasticity had a negligible effect on the size of the primary drop produced. The authors concluded that even though the presence of elasticity created a significant difference in drop formation (necking) dynamics, this contributed little to the final drop size and drop production rate. Starting out from the observation of Husny et al. and performing analyses of filament self-thinning in the Boger dispersed phase, Arratia et al. measured polymer extensional viscosity [22,23]. Lee et al. provided quantitative insights on filament formation and self-thinning in the case of non-Newtonian dispersed phase and surfactants added in the continuous phase [24]. They found the necking process was more rapid in the case without surfactant or viscoelasticity. However, if either surfactant or viscoelasticity was added to the dispersed-phase liquid, the necking rate was slowed. Sang et al. [25] numerically simulated droplet breakup in a T-junction for power-law and Bingham fluids. With the increase of the capillary number of continuous phases based on effective viscosity, a laminar segment was formed in the T-junction before droplet separation. For Bingham fluids, droplets were not spherical. The droplet extension increased with the increase of the yield stress. Roumpea et al. found that plug lengths in the systems with non-Newtonian continuous phase were larger than in the Newtonian one and increased with the xanthan gum concentration, which is responsible for fluid shear-thinning behavior [26]. Linear coefficients in the Garstecki formula [27] for plug length were different for various concentrations, indicating an influence of non-Newtonian solutions.

Currently available data show that there is a lack of flow-pattern-map unification in the case of non-Newtonian fluids. Moreover, to our best knowledge, there are no flow-pattern studies for cases when non-Newtonian fluids act as a dispersed phase. It is tacitly accepted that the shear rate in the continuous phase has a crucial role in the flow-pattern formation. Nevertheless, it still has to be proven, and the influence of the non-Newtonian dispersed phase on flow-pattern maps should be studied in more detail. Therefore, here we pay attention to the dispersed phase in which viscosity depends on the shear rate. To date, the influence of the non-Newtonian dispersed phase on the plug length is still not clear. Finally, when discussing non-Newtonian fluids, the assessment of shear rates in the systems is another key question. In the literature presented in the survey, the authors used at least four different approaches for shear-rate calculation, and the resulting values can differ by orders of magnitude (see Section 3.1). The choice of an optimal model and its application to the description of flow-pattern transition in a two-phase microfluidic flow is an important problem that restricts the design and applications of microchannels.

In this paper, we address the questions of flow-pattern-map unification, the plug-formation mechanism, and correct estimation of shear rates in the case of non-Newtonian fluid flows in microchannels. The dispersed phase was chosen to be non-Newtonian because there is a lack of data on flow-pattern maps and plug formation for such systems. In contrast to many other research works, we utilized non-Newtonian fluids in conjunction with Newtonian ones with different viscosities to elucidate the influence of viscosity ratio and shear-thinning separately. Different models for shear rate were compared and analyzed for applicability in the flow-pattern-map unification. For the first time, the influence of the non-Newtonian dispersed phase on the transition from segmented to continuous flow was shown and quantitatively described. The mechanism of plug formation appeared to be dependent on the shear-thinning of the dispersed fluid when independent of the viscosity ratio.

## 2. Materials and Methods

**Figure 1:**
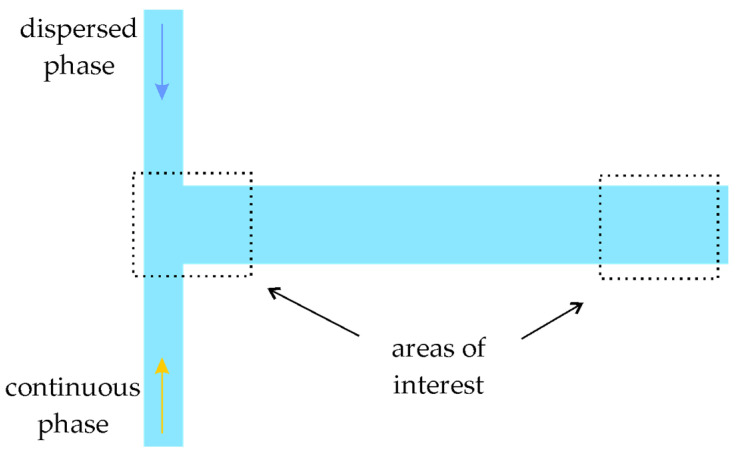
The scheme of the T-shaped microchannel.

T-shaped microchannels with rectangular cross-sections were fabricated using polymethylmethacrylate (PMMA) material by micromilling technique with 1 µm accuracy by ChipShop (Germany). The scheme of the microchannels is presented in Figure 1. The dimensions of the inlet channels were 200 × 200 µm^2^ with an 11.5 mm length, and the dimensions of the outlet channel were 200 × 400 µm^2^ with a 22.5 mm length. The flow of continuous and dispersed phases was organized as presented in Figure 1.

In order to reveal the influence of shear-thinning behavior of the dispersed phase on the liquid–liquid flow, three non-Newtonian liquids were used: xanthan gum (Sigma Aldrich) aqueous solution at a concentration of 0.5% (*w*/*w*), and sodium carboxymethyl cellulose (CMC, Sigma Aldrich, average M_w_ ~ 250,000, degree of substitution 0.7) aqueous solutions at concentrations of 0.5% (*w*/*w*) and 1.5% (*w*/*w*). Aqueous solutions of the shear-thinning fluids were prepared by continuously pouring the powders into distilled water and stirring for approximately 20 min until complete dilution. Three aqueous glycerol solutions at different concentrations, further referred to as G1, G2, and G3, were used as Newtonian dispersed phases in order to perform comparative studies of flow-pattern boundaries and slug-flow features. The continuous phase was always castor oil in the experiments with both Newtonian and non-Newtonian dispersed phases.

The physical properties of the liquids presented in Table 1 were measured at 23 °C. The density ρ was measured by weighing the known volume of fluid with a relative error of 5%. Liquid–liquid interfacial tension σ was measured with 0.5 mN/m accuracy by a pendant drop method using the KRUSS DSA-100 setup. The static contact angles θ were measured by a sessile drop technique: a drop of the dispersed phase was placed on the PMMA substrate immersed in the continuous liquid. The multiple measurements of contact angle at different places of substrate gave a standard deviation within 4°.

**Figure 2:**
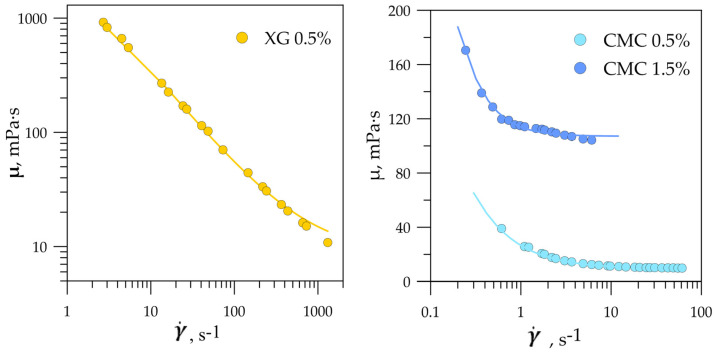
Dynamic viscosity of shear-thinning liquids depending on shear rate.

The dynamic viscosity of liquids µ was measured using a rotational viscosimeter with coaxial cylinders. During the measurements, the liquid was thermostated at 23 ± 0.1 °C. The relative errors of the dynamic viscosity measurements were within 3% and 4% for Newtonian and non-Newtonian liquids, respectively. The dependences of the measured dynamic viscosity of the shear-thinning liquids on the applied shear rate are presented in Figure 2. Over the range of applied shear rates, the aqueous solution of xanthan gum exhibited strong shear-thinning behavior. The dynamic viscosity of the fluid changed by two orders of magnitude, with a variation in the shear rate from 2.7 to 1312 s^−1^. The dynamic viscosity of the CMC aqueous solutions rapidly decreased at low shear rates, further showing the properties of a Newtonian liquid.

To approximate the dependence of viscosity on shear rate, the Bird–Carreau model was applied [28]:

(1) $$µ={µ}_{\infty }+\left({µ}_{0}-{µ}_{\infty }\right)\cdot {\left[1+{\left(k\dot{\gamma }\right)}^{2}\right]}^{\frac{n-1}{2}}$$

where ${µ}_{0}\;$is the dynamic viscosity at zero shear rate, ${µ}_{\infty }$ is the dynamic viscosity at an infinite shear rate, k is the relaxation time, and n is the power index. For nonlinear approximation, the Levenberg–Marquardt algorithm was used. The coefficient of determination R^2^ was 0.99 for the xanthan gum solution and 0.96 for the CMC solutions. The values of approximated parameters are presented in Table 2.

A study of the degradation of the resulting solutions over time was carried out. For this, the physical properties of liquids were measured immediately after the preparation of the solution, after 24 h, and after 10 days. It was found that the physical properties of liquids varied within the measurement error.

Flow rates of dispersed and continuous phases were controlled by a double syringe pump (Gemini 88, KD Scientific) with a relative accuracy of 0.35%. A Zeiss AxioObserver.Z1 inverted microscope with a 5× magnification lens and a mounted halogen lamp was used to visualize the flow. Flow images were recorded by a high-speed CMOS camera (pco.1200 hs) with a frame rate up to 500 Hz. Flow patterns were visualized in transmitted light due to the refractive index difference of continuous and dispersed phases in the T-junction zone, and 15 mm away from the T-junction. The resolution of the flow images was 2.7 µm/pix. For each flow rate of the continuous and dispersed phases, 500 images were recorded. The reproducibility of the experiments was checked by ensuring the identical flow regimes at specified superficial velocities and the flow properties, such as slug length and velocity.

## 3. Results and Discussion

### 3.1. Flow Patterns

**Figure 3:**
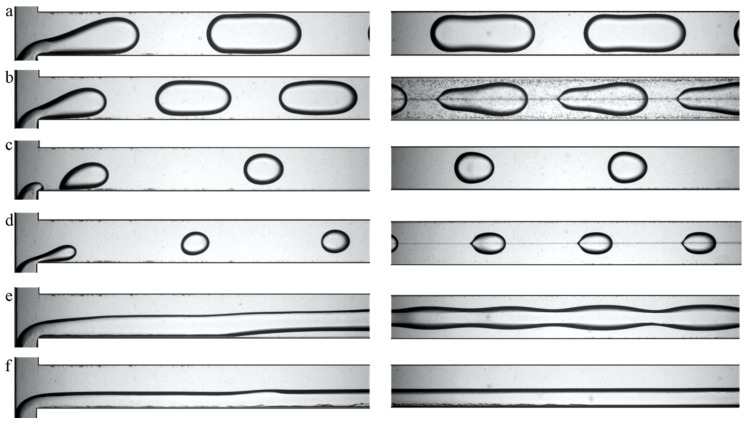
Typical flow patterns for the flow of CMC 0.5%–castor oil at a T-junction and the end of microchannel: (**a**) slug flow, (**b**) slug flow with microdroplets, (**c**) droplet flow, (**d**) droplet flow with microdroplets, (**e**) rivulet flow, and (**f**) parallel flow.

High-speed visualization of the immiscible liquid–liquid flow was performed in a wide range of superficial velocities of dispersed and continuous phases from 29 µm/s to 2300 µm/s. The typical flow patterns that were revealed are presented in Figure 3, in which flow pictures for CMC 0.5%–castor oil are presented as an instance. At a low flow rate of the dispersed phase, slug flow was observed (Figure 3a). At a high flow rate of the continuous phase and a low flow rate of the dispersed phase, the droplet flow was realized, with a droplet diameter less than or equal to the channel width (Figure 3c). An increase in bulk velocity led the microdroplets to break off from the rear of the slug or droplet due to the formation of V-shaped contact lines on the top and bottom channel walls (Figure 3b,d). This phenomenon was observed previously in [29] for flows of liquids with low viscosity ratio λ = µ_d_/µ_c_. With a further increase in the bulk velocity, for high flow rates of the dispersed phase and low flow rates of the continuous phase, we observed a rivulet regime with wavy boundaries (Figure 3e). In this regime, the rivulet of the dispersed phase flowed along the upper and/or lower walls of the microchannel. At the highest available bulk velocities, a parallel flow was established, in which the dispersed phase also moved along the side wall of the channel, thus wetting three walls (Figure 3f).

The main difference in the flow patterns for XG–castor oil from the previous set of fluids was the presence of a rivulet regime with parallel boundaries. For a set of CMC 1.5%–castor oil, the rivulet flow was not found in the range of the investigated superficial velocities. High-speed visualization of the flow of the castor oil–G1 aqueous glycerol solution made it possible to elicit the following flow regimes: slug flow, slug flow with microdroplets, droplet flow, droplet flow with microdroplets, rivulet, and parallel flow. For the G1 and G2 aqueous glycerol solutions and castor oil, flow, slug, droplet, and parallel flow were observed. Since these liquid–liquid sets do not feature a low viscosity ratio λ, flow regimes with microdroplet break-off from the rear meniscus of the slugs and droplets were not recorded.

**Figure 4:**
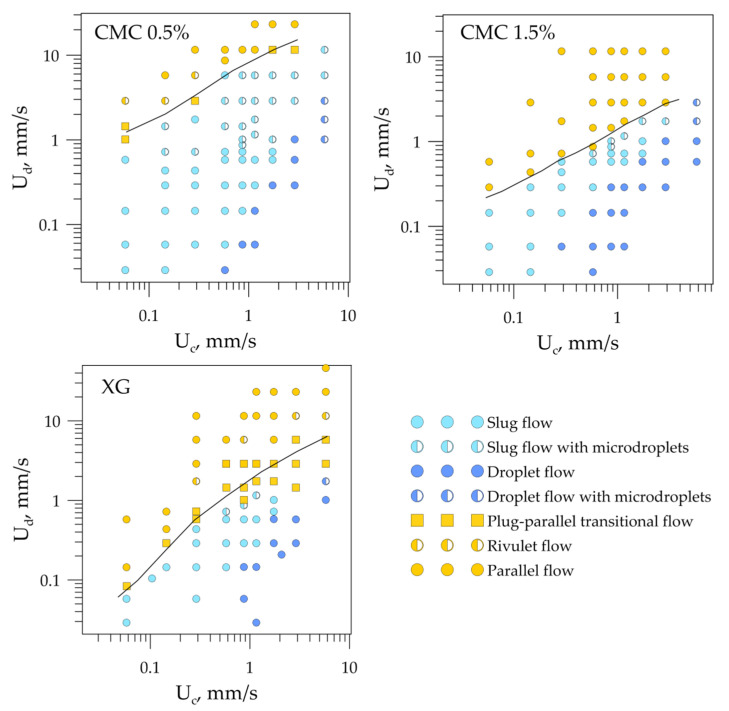
Flow-pattern maps for liquid–liquid flows when the dispersed phase was non-Newtonian.

Flow-pattern maps were constructed based on the high-speed visualization of flow regimes for all six sets of immiscible fluids. Flow-pattern maps for the cases when the dispersed phase was non-Newtonian are presented in Figure 4 in terms of superficial velocities U_c_ and U_d_ of the continuous and dispersed phases. One can see that the flow regimes’ mutual arrangement on all three flow-pattern maps was the same. However, the boundaries between the flow regimes were shifted relative to each other along the axis representing the dispersed phase superficial velocity.

**Figure 5:**
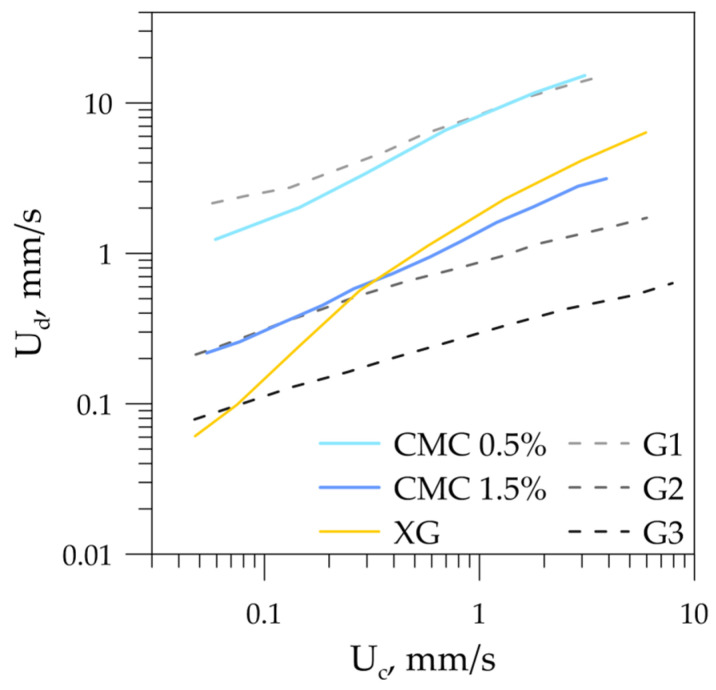
Boundaries between segmented and continuous-flow rates for liquid–liquid flows with Newtonian and non-Newtonian dispersed phases.

One of the important tasks in developing microfluidic devices using immiscible liquids is to determine the boundaries between segmented (slug, droplet) and continuous (parallel, rivulet, annular) flow regimes. For example, when carrying out liquid–liquid extraction in microchannels, in most cases, it is optimal to operate in slug or droplet flow regimes—the circulation of velocity inside the slugs intensifies the mass transfer, which increases the extraction efficiency. However, in the presence of surfactants or nanoparticles in the system, the separation of substances after extraction in the slug regime is largely difficult; therefore, continuous operation, in this case, is favorable. In order to compare the boundary position between continuous and segmented flow patterns for all liquid–liquid sets with Newtonian and non-Newtonian dispersed phase, they were drawn on one plot (Figure 5). As expected, the boundaries drawn in terms of superficial velocities of the dispersed and continuous phases did not coincide. When comparing the boundaries, one can see that they are shifted by one order of magnitude for G1 and G2, and by half an order of magnitude for G2 and G3. Continuous-flow regimes prevailed for an aqueous glycerol solution with a higher viscosity (G3) due to the energy dissipation into viscous friction, which prevented forming a new interface (slugs and droplets). Thus, the boundary between segmented and continuous-flow regimes lay at lower superficial velocities of the dispersed phase. A decrease in the viscosity of aqueous glycerol solutions led to an increase in the dispersed-phase superficial velocity, at which segmented flow regimes were realized. It should be noted that the boundaries between segmented and continuous flow regimes had the same slope (parallel to each other) for all three Newtonian dispersed phases. For the liquid–liquid flows with non-Newtonian dispersed phase, the boundaries were not parallel to each other or to those of the Newtonian dispersed phase cases. At low flow rates of phases (low shear rates), the viscosity of the XG was very high, and energy was spent on viscous friction; thus, continuous regimes prevailed on the flow-pattern map. With an increase in the flow rates of the phases, the effective dynamic viscosity of the shear-thinning liquid decreased, and the curve of the separation of the flow regimes bent. The boundary between flow regimes for CMC 0.5% and CMC 1.5% behaved in a similar way. In the case of these non-Newtonian fluids, the curves deviated from the straight line to the least extent and lay near the boundaries for the aqueous glycerol solution G1 and G2. This was due to the smallest variation in the effective viscosity of the given non-Newtonian fluids compared to XG (see Table 1). Thus, it can be concluded that the dynamic viscosity of the dispersed phase was the governing parameter that affected the flow regimes of the immiscible liquids.

**Figure 6:**
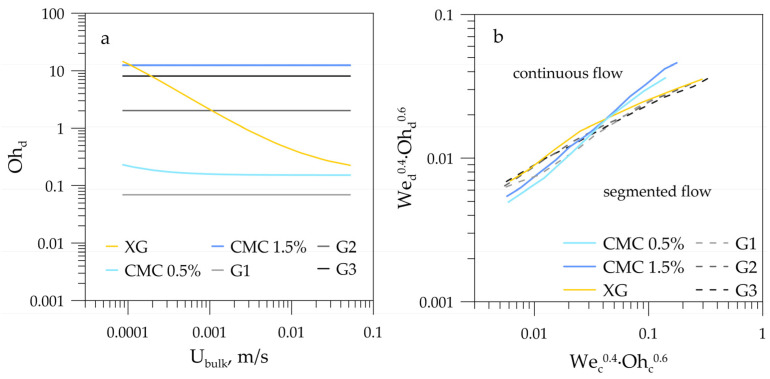
(**a**) Ohnesorge numbers for Newtonian and non-Newtonian dispersed phases as a function of bulk velocity; (**b**) boundaries between segmented and continuous-flow rates for liquid–liquid flows with Newtonian and non-Newtonian dispersed phases in terms of the proposed nondimensional complex.

To quantitatively determine the effect of the viscosity of liquids on the flow-regime maps, the dependencies of the Ohnesorge number (Oh) for each dispersed phase used in the experiment on the bulk velocity were plotted (Figure 6a). The physical meaning of the Ohnesorge number can be obtained by multiplying its numerator and denominator by the square of the channel hydraulic diameter and the bulk velocity:

(2) $$Oh=\frac{µ}{\sqrt{D_{h}\sigma \rho }}=\frac{µuD_{h}^{2}}{\sqrt{D_{h}^{3}\sigma \rho u^{2}}}=\frac{µ\frac{u}{D_{h}}D_{h}^{2}\cdot D_{h}}{\sqrt{\left(\rho u^{2}D_{h}^{3}\right)\cdot \left(\sigma D_{h}^{2}\right)}}$$

The numerator contains energy dissipation due to viscous forces, and the denominator is the geometric mean of the kinetic energy of the system and the surface energy. The lower the Ohnesorge number, the weaker the frictional losses due to viscous forces, i.e., most of the energy is converted into creating a new surface due to surface-tension forces, and a drop is formed. At high values of the Ohnesorge number, all the energy of the system dissipates due to viscous forces, and drops are not formed. As the Ohnesorge number is directly proportional to the dynamic viscosity of the liquid, it is essential to estimate the last properly. In non-Newtonian fluids, the dynamic viscosity is shear-dependent, and the determination of corresponding shear rates in multiphase systems is a distinct task. There are several main approaches that are usually utilized by researchers. The first and the most common one is to assess effective viscosity and effective shear rates considering Poiseuille flow in a pipe of, for example, power-law fluid [30]. This approach gives an effective shear rate as (8U_avg_/D_h_)*f(n), where f(n) is a certain function of the fluid power-law index, U_avg_ is an average velocity, and D_h_ is an equivalent diameter, which coincides with the cross-section diameter in the case of the circular pipe. In this work, we used this approach to consider the influence of fluid bulk velocity on the viscosity of non-Newtonian dispersed phase, and took U_avg_ = U_bulk_. Neglecting the influence of power-law index n on the shear rate, thus assuming f(n) equal to unity in this formula for effective shear rate, and letting U_avg_ = U_c_, one can obtain a Spisak model for shear-rate estimation, which is frequently used in gas–liquid flows. The Spisak approach to viscosity estimation in conjunction with power-law fluid models can be found in [18,19]. Roumpea et al. [26] calculated effective capillary numbers in their work using a model by Linder et al. [30], proposed for Hele–Shaw geometry and purportedly a good approximation in the case of strong shear-thinning behavior of power-law fluid (*n* < 0.65). Chiarello et al. assessed shear rates based on the averaging of the parabolic velocity profile, and introduced an effective capillary number derived from the comparison of differential equation terms. This approach was shown to be very effective in the description of plug formation for non-Newtonian fluids. Another example is a simple evaluation of shear rate as U_c_/D_h_, applied together with the Bird–Carreau model in [31]. The key notes of shear-rate estimations by various authors are summarized in Table 3.

The difference in non-Newtonian viscosity estimated using listed models can achieve an order of magnitude for low shear rates in strongly shear-thinning fluids, e.g., the XG solution in the current work. Here, we used the approach described above with a shear-rate estimation of 8U_bulk_/h, where h is the smallest dimension of the microchannel, i.e., its height. Since the flow rates of both phases influence the flow pattern transitions, the bulk velocity U_bulk_ is used as a characteristic velocity value instead of the superficial velocity of a single phase U_c_ or U_d_. The necessity of considering the impact of flow rates from both phases is evident from experiments and indicated by the nonzero slope of the transition boundaries (see Figure 5). The term f(n) that appears for power-law fluids introduces difficulties in the case of shear-thinning fluids, which are not described well by a power-law approximation such as for the CMC solutions used. Therefore, we also neglected the influence of this term on the shear rate, supposing it to be equal or close to unity (the power-law approximation gives n = 0.24 and f(n) = 1.15 for the XG solution).

Our previous studies of the viscosity influence on the flow-pattern map of immiscible liquid–liquid flows in a T-shaped microchannel [17] let us conclude that the nondimensional complex We^0.4^·Oh^0.6^ allows the construction of a universal flow-pattern map that takes into account all properties of the liquids. Thus, here we employed this complex to plot the boundary between segmented and continuous-flow patterns for the liquid–liquid flows with Newtonian and non-Newtonian dispersed phases (Figure 6b). The agreement between the boundaries for both the Newtonian and non-Newtonian dispersed phases proved that the applied shear-rate estimation to calculate the effective Ohnesorge number was correct. All other models presented in Table 1 underpredicted the effective shear rates in the case of segmented-to-continuous-flow pattern transition. The reason for this is possibly a significant difference in the local shear rates for various flow patterns. Thus, the models appropriate for the slug formation, in which only the continuous phase superficial velocity U_c_ is considered, failed to predict the transition from slug to parallel-flow regimes.

**Figure 7:**
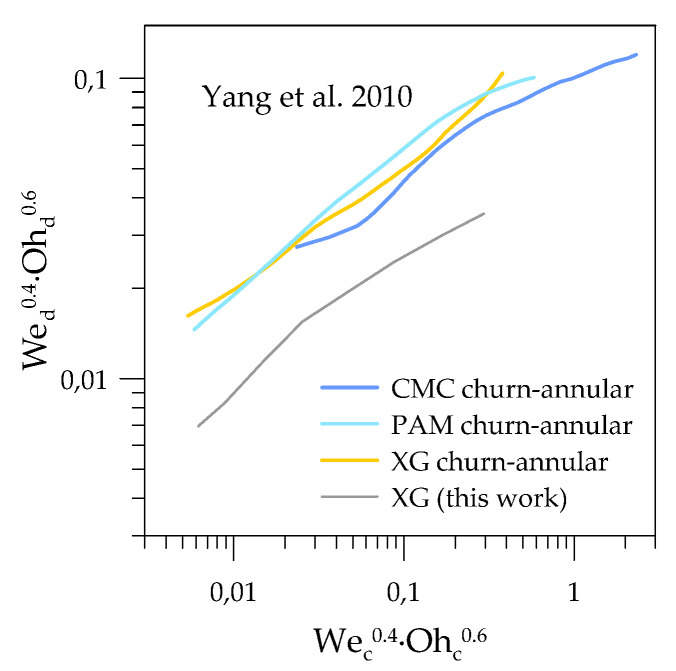
Comparison of the segmented-to-continuous-flow pattern transition for Yang et al. [18] and the current work in the unified dimensionless coordinates.

A comparison with available literature data on non-Newtonian fluid flows in microchannels was performed in order to test an assumption about effective viscosity dependence on the bulk velocity with regard to flow-pattern transition from segmented to continuous flow. We used data from Yang et al. [18] for non-Newtonian solutions of XG, CMC, and PAM in gas–liquid flows. An effective viscosity was calculated using a power-law model with values of n and k provided by the authors and using a shear rate estimated as 8U_bulk_/h. The resulting unification of flow-pattern boundaries is presented in Figure 7, in which the boundary for XG solution from current work is presented as a reference. One can see that the proposed shear-rate estimation gave a reasonable unification of transition boundaries for different non-Newtonian solutions. The comparison with our results showed a similar slope of the transition lines, indicating that the mechanism of flow pattern transition was the same, and the churn-to-annular transition from Yang et al. can be considered as a segmented-flow/continuous-flow boundary. Finally, the mismatch in the absolute values may be explained by the influence of the channel-inlet configuration or the aspect ratio, which were not taken into account in the proposed dimensionless complex. In addition, channel-wall wettability could also have influenced the two-phase flow-pattern boundaries in the microchannels, since adhesion forces were not considered in the proposed nondimensional complex.

### 3.2. Slug Flow

**Figure 8:**
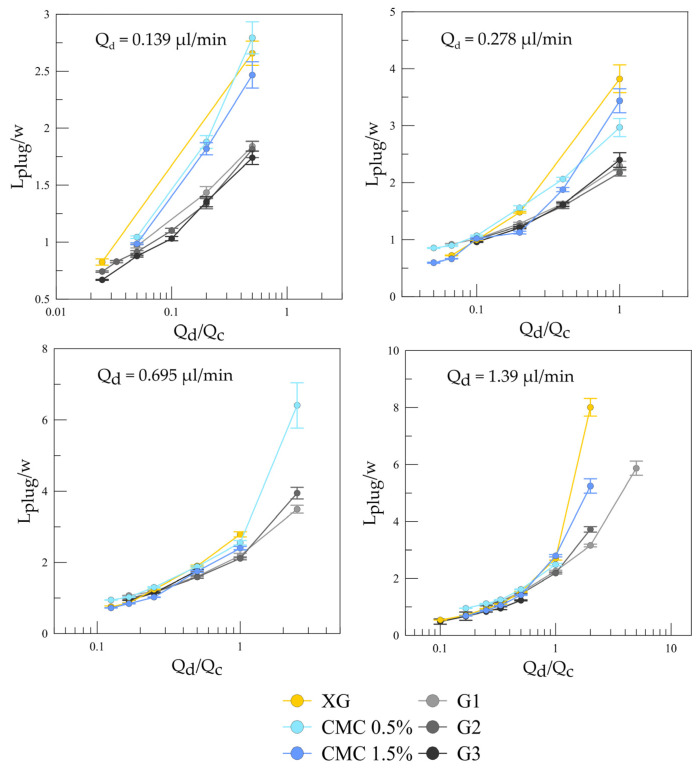
Slug length depending on flow-rate ratio at different dispersed-phase flow rates for the Newtonian and non-Newtonian dispersed phases.

**Figure 9:**
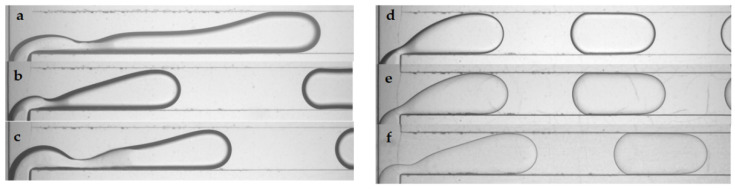
Slug formation of Newtonian and non-Newtonian dispersed phases: (**a**) XG, (**b**) CMC 0.5%, (**c**) CMC 1.5%, (**d**) G1, (**e**) G2, and (**f**) G3.

In order to reveal the influence of the shear-thinning dispersed phase on the slug flow, slug length was measured for all liquid–liquid sets and compared with the cases of the Newtonian dispersed phase. Slug length was measured at the end of the microchannel using DMV video-processing software [33]. The processing steps included background subtraction and slug-edge detection, with subsequent filling of the inner part of the slugs. For each flow rate of the dispersed and continuous phases, the averaging was performed for 20–30 slugs. The measurement uncertainty of the slug length did not exceed 5.4 µm. The slug-length plots normalized by the microchannel width depending on the flow rate ratio of the dispersed and continuous phases at different flow rates of the dispersed phase are presented in Figure 8. Comparison of the plots allowed us to conclude that at lower flow rates of the non-Newtonian dispersed phase, the increase of the flow rate ratio led to the considerable growth of the slug length, in contrast to the case of the Newtonian dispersed phase. This discrepancy occurred at a low flow-rate ratio and the low flow rate of the dispersed phase. The higher the flow rate of the dispersed phase, the higher the flow rate ratio at which deviation from Newtonian dispersed phase case occurred. Slug formation at the T-junction was examined in detail for the Newtonian and non-Newtonian dispersed phases. The pictures of slug formation at Q_d_ = 0.278 µL/min, Q_c_ = 0.278 µL/min are presented in Figure 9. The high dynamic viscosity of the dispersed phase at small flow rates resisted the slug break-off at the entrance of the mixing channel, which prompted the dispersed phase to flow further downstream, and the slug-formation mechanism changed to the jetting regime. An increase in the dispersed-phase flow rate decreased the effective dynamic viscosity of the non-Newtonian liquid, and the discrepancy in slug formation vanished.

## 4. Conclusions

An influence of the shear-thinning feature of the dispersed phase on the flow of immiscible liquids in a T-shaped microchannel was studied based on high-speed visualization. Three non-Newtonian solutions with different rheological characteristics were used as a dispersed phase, while three aqueous glycerol solutions with different viscosities were used as Newtonian dispersed phases to conduct a comparative study. Castor oil was always a continuous phase in the experiments. Typical flow patterns were visualized in a wide range of superficial velocities of the dispersed and continuous phases. Flow-patterns maps were drawn for all liquid–liquid sets. The following concluding observations can be highlighted:

1. The boundaries between segmented and continuous-flow patterns drawn on the flow-pattern maps in terms of superficial velocities of the phases were shifted relative to each other for the cases of shear-thinning and Newtonian dispersed phases with different viscosities. While the boundaries for Newtonian liquids were parallel to each other, the boundaries for the case of the non-Newtonian dispersed phase were not.
2. The most appropriate model of average shear-rate estimation based on bulk velocity was chosen and applied to evaluate an effective dynamic viscosity of a shear-thinning fluid.
3. The nondimensional complex We^0.4^·Oh^0.6^ could be successfully utilized for universal flow-pattern-map construction for both Newtonian and non-Newtonian dispersed phases, for which the Ohnesorge number was calculated using an effective viscosity based on the average shear rate in a microchannel.
4. Comparison with the experimental data from literature showed that the proposed nondimensional complex We^0.4^·Oh^0.6^ unified flow-pattern boundaries when the continuous phase exhibited non-Newtonian properties.
5. The shear-thinning dispersed phase influenced the slug-formation mechanism and slug length. At low flow rates of the dispersed and continuous phases, a jetting regime of slug formation was established, leading to a dramatic increase in slug length.

## Figures and Tables

**Table 1:** Physical properties of the liquids used in the experiments @ 23 °C.

Physical Property	Castor Oil	CMC 0.5%	CMC 1.5%	XG 0.5%	G1	G2	G3
ρ, g/cm^3^	0.962	0.99	0.99	0.99	1.128	1.213	1.226
σ, mN/m	-	14.6	13.7	10.7	15	12.8	11.97
θ, °	-	159.3	151.9	165.3	168.4	167	168.5
µ, mPa·s	760	9.4–14.3	107.1–108	29–1232	4.7	130	506

**Table 2:** The parameters of the Bird–Carreau model for non-Newtonian liquids.

Parameter	CMC 0.5%	CMC 1.5%	XG 0.5%
${µ}_{0}$, mPa∙s	200	372.9	1313
${µ}_{\infty }$, mPa∙s	9.44	107.1	8.7
k, s	10.68	8.9	0.457
n	−0.014	−0.67	0.129

**Table 3:** Different approaches to shear-rate estimation in two-phase microfluidic flows.

Authors	$\mathrm{Shear}-\mathrm{Rate}\;\mathrm{Estimation}\;\dot{\gamma }$	Comments on the Estimation of Effective Viscosity
Zhang et al. [19] and Yang et al. [18]	$\dot{\gamma }=\frac{8U_{c}}{D_{h}}$	$\mathrm{Power}-\mathrm{law}\;\mathrm{fluid}\text{:}\;\mu_{eff}=k{\dot{\gamma }}^{n-1}$
Roumpea et al. [26]	$\dot{\gamma }=\frac{2U_{c}\left(1+2n\right)}{D_{h}\left(1+n\right)}$	$\mathrm{Power}-\mathrm{law}\;\mathrm{fluid}:\;\mu_{eff}=k{\dot{\gamma }}^{n-1}$
Chiarello et al. [32]	$\dot{\gamma }=\frac{3U_{c}}{\delta }$	Here *δ* is a smallest size of a rectangular channel cross-section. The authors introduced effective capillary number Ca’ = n Ca(µ_eff_)
Fu et al. [20]	$\dot{\gamma }=\frac{U_{c}}{D_{h}}$	Viscosity was calculated using the Bird–Carreau model (Equation (1))

## Nomenclature

**Symbol**	**Formula**	**Description**
D_h_, m		hydraulic diameter
w, m		microchannel width
U, m/s		superficial velocity
U_bulk_, m/s		bulk velocity
Q, m^3^/s		flow rate
µ, Pa·s		dynamic viscosity
σ, N/m		interfacial tension
ρ, kg/m^3^		density
θ, °		contact angle
Ca	$\frac{\mu U}{\sigma }$	capillary number
Oh	$\frac{\mu }{\sqrt{D_{h}\sigma \rho }}$	Ohnesorge number
Re	$\frac{\rho UD_{h}}{\mu }$	Reynolds number
We	$\frac{\rho U^{2}D_{h}}{\sigma }$	Weber number
d		dispersed phase
c		continuous phase
